# Prediction of risk of ischemic heart disease in first-diagnosed systemic lupus erythematosus patients in taiwan: is air pollution exposure a risk factor?

**DOI:** 10.1186/s41927-023-00337-8

**Published:** 2023-06-08

**Authors:** Pei-Yun Chen, Yu-Tse Tsan, Chao-Tung Yang, Yun-Mei Lee, Li-Li Chen, Wen-Chao Ho, Shu-Hua Lu

**Affiliations:** 1 Departmant of public Health, China Medical University, Taichung, Taiwan; 2 Jen-Teh Junior College of Medicine, Nursing and Management, Miaoli, Taiwan; 3 Division of Occupational Medicine, Department of Emergency Medicine, Taichung Veterans General Hospital, Taichung, Taiwan; 4 Department of Occupational Safety and Health Office, Taichung Veterans General Hospital, Taichung, Taiwan; 5 School of Medicine, Chung Shan Medical University, Taichung, Taiwan; 6 Department of Computer Science, Tunghai University, Taichung, Taiwan; 7 Department of Nursing, Cardinal Tien Junior College of Healthcare and Management, New Taipei City, Taiwan; 8 School of Nursing, China Medical University, Taichung, Taiwan; 9 Department of Nursing, China Medical University Hospital, Taichung, Taiwan

**Keywords:** Ischemic heart disease, Systemic lupus erythematosus, Air pollution, Hazard

## Abstract

**Background:**

Air pollution is a key public health factor with the capacity to induce diseases. The risk of ischemia heart disease (IHD) in those suffering from systemic lupus erythematosus (SLE) from air pollution exposure is ambiguous. This study aimed to: (1) determine the hazard ratio (HR) of IHD after the first-diagnosed SLE and (2) examine the effects of air pollution exposure on IHD in SLE for 12 years.

**Methods:**

This is a retrospective cohort study. Taiwan’s National Health Insurance Research Database and Taiwan Air Quality Monitoring data were used in the study. Cases first diagnosed with SLE in 2006 cases without IHD were recruited as the SLE group. We randomly selected an additional sex-matched non-SLE cohort, four times the size of the SLE cohort, as the control group. Air pollution indices by residence city per period were calculated as the exposure. Life tables and Cox proportional risk models of time-dependent covariance were used in the research.

**Results:**

This study identified patients for the SLE group (n = 4,842) and the control group (n = 19,368) in 2006. By the end of 2018, the risk of IHD was significantly higher in the SLE group than in the control group, and risks peaked between the 6th and 9th year. The HR of incidence IHD in the SLE group was 2.42 times that of the control group. Significant correlations with risk of developing IHD were noted for sex, age, CO, NO_2_, PM_10_, and PM_2.5_, of which PM_10_ exposure had the highest risk of IHD incidence.

**Conclusions:**

Subjects with SLE were at a higher risk of IHD, especially those in the 6th to 9th year after SLE diagnosis. The advanced cardiac health examinations and health education plan should be recommended for SLE patients before the 6th year after SLE diagnosed.

## Introduction

Ischemic heart disease (IHD) is a major cause of mortality worldwide. In total, 20,457 individuals died of IHD in 2020, more than half of which were men (11,809 individuals) and frequently the primary breadwinners for their households [1]. IHD is a serious disease that entails a high probability of sudden death. Hence, families are often caught unprepared, and the effects on the family and society are profound.

Although some IHD-related risk factors cannot be modified, others can be mitigated if a high-risk patient is identified in time to devise a prevention strategy. Studies have indicated that the leading cause of IHD is atherosclerosis of coronary arteries and diminished blood supply, which damages the myocardium [2]. However, recent literature has reported that atherosclerosis itself is a chronic inflammatory condition [3]. The initial pathophysiological mechanism of IHD begins with atherosclerosis, which involves low-grade inflammation of the arterial intima (inner layer). The disease may persist for decades before it progresses to clinical symptoms. This low-grade state of inflammation induces thickening of the coronary artery lining, eventually resulting in varying degrees of arterial lumen stenosis [4]. The chronic inflammation of atherosclerosis promotes the retention of cholesterol-rich low-density lipoprotein (LDL) particles in the arterial wall. Subsequently, oxidative modification of vascular LDL particles promotes an inflammatory response to this endothelial injury. Macrophages ingest the LDL particles and develop into lipid-foam cells.

Further, local inflammation is stimulated. Early atherosclerotic lesions called ‘fatty streaks’ are composed of lipid deposits, cholesterol-laden macrophages, foam cells, and T cells. With disease progression, immune cells interact with resident blood vessel wall cells, eventually forming atherosclerotic plaques [3]. Finally, atherosclerotic plaques are composed of inflammatory cells, cell debris, smooth muscle cells, and cholesterol [4]. This inflammatory-related perspective differs from the previous understanding of the risk factors for IHD. It is therefore desirable to determine whether inflammation-related diseases are associated with IHD.

Inflammation-related diseases include systemic lupus erythematosus (SLE), diabetes, hypertension, and hyperlipidemia. The SLE-mediated relationship between body image and fatigue severity significantly influences a patient’s psychological condition and quality of life (QOL) [5]; as the state of SLE deteriorates, patients’ medical expenses and work disability increase [6, 7]. The diverse symptoms of SLE, such as fatigue severity, impaired body image, depression, and anxiety, impact the individual’s QOL, and not only in the psychological domain [5]. The symptoms associated with SLE can significantly affect patients’ QOL in terms of work and social life [8]. SLE results from a malfunctioning immune system that attacks self-antigens [9]. The actual etiology of SLE remains unknown. Numerous risk factors are associated with immune-related diseases. A complex interaction likely occurs among genetic factors, infectious agents, ultraviolet rays, smoking, and hormonal factors, leading to immune system disturbance and disease manifestation [10]. SLE-related risk factors continue to be investigated.

Even though healthcare quality has improved for SLE and IHD in the last century, SLE progression to IHD is still unavoidable. The major difference between today and the previous century is air pollution.

This study aimed to: (1) determine the hazard ratio (HR) of IHD after the first-diagnosed SLE and (2) examine the effects of air pollution exposure on ischemic heart disease (IHD) in Systemic lupus erythematosus (SLE) for 12 years. We used data from the National Health Insurance Research Database (NHIRD) and Taiwan Air Quality Index data (AQI) of Taiwan to investigate IHD development in the population newly diagnosed with SLE from 2006 to 2018.

## Methods

### Study Design and Data source

This study adopted a longitudinal cohort design over 12 years and involved a secondary analysis based on data from the NHIRD of Taiwan. The NHIRD is the largest medical database in Taiwan and contains a wealth of health insurance information. National Health Insurance has covered 99.82% of individuals in Taiwan since 1996. Enrolment is compulsory for most of the population. Therefore, the data can be considered to accurately represent the situation of the population in Taiwan. Medical records, pharmaceutical information, admission and outpatient data, and physical examination records are included in the NHIRD. This study was approved by the Institutional Review Board of China Medical University and Hospital in Taiwan [CMUH111-REC3-040].

### Comorbidities and medications

Diseases related to inflammation, including diabetes, hypertension, and hyperlipidemia, were considered potential confounders. Variables (i.e., age and sex) that influenced both SLE and IHD were defined as confounders.

### Sampled study cohorts and data Collection

The study population data were extracted from the NHIRD. The inclusion criteria for the study participants were a new diagnosis of SLE in 2006 and an age above 20 years. The exclusion criteria were a diagnosis of SLE or IHD before 2006. We randomly selected an additional sex-matched non-SLE cohort, four times the size of the SLE cohort, as the control group. The SLE disease codes were identified based on the International Classification of Diseases, Ninth Revision (ICD-9) codes 710. IHD includes angina, acute myocardial infarction, and other IHDs. The IHD disease codes were identified based on the ICD-9 codes 410–414.

**Fig. 1:**
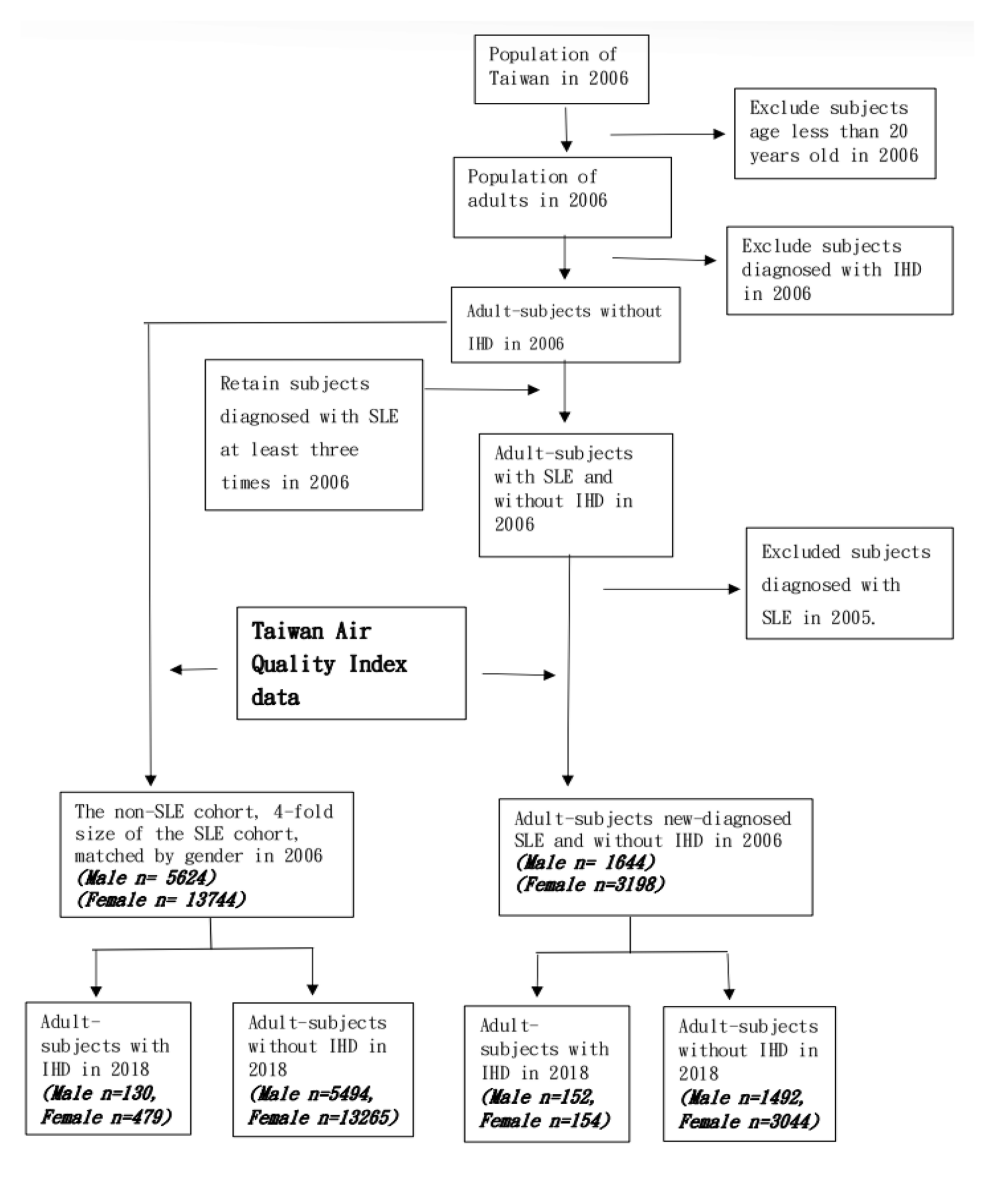
Flow diagram of the sampling process SLE: Systemic lupus erythematosus IHD: Ischemic heart disease

This research focused on adults. SLE and IHD are considered chronic diseases, and subjects with these diseases should visit a doctor at least once a year. Therefore, subjects diagnosed with SLE in 2005 or earlier were excluded. The 4,842 subjects newly diagnosed with SLE and without IHD in 2006 were defined as the “case group” and matched with 19,368 subjects for the control group. We assumed that exposure to air pollution was similar for all subjects in a given area. Patients were linked to the nearest AQI station via their residence, which was used to define their air pollution exposure. We used the residence of patients of both groups to concatenate the data of NHIRD and AQI for subsequent statistical analysis. (Fig. 1).

### Statistical analysis

SAS version 9.4 was used to analyze the data. The chi-square test was used to identify differences in patient characteristics by SLE incidence. Life-table method analysis with log-rank tests was performed to examine differences in SLE incidence between male and female patients. Cox proportional hazard regression analyses were performed to analyze the HR of IHD in the SLE and control groups.

## Results

Demographical data of subjects in 2006, IHD incidence, and air pollution condition in 2018 are summarized in Table 1. This cohort consisted of 24,210 participants. The study included 4,842 subjects (male n = 1,644, female n = 3,198) with first-diagnosed SLE (SLE group) and 19,368 participants (male n = 5,624, female n = 13,744) without SLE (control group). More than half of the SLE subjects were female. The number of subjects suffering from SLE was higher in the “above 60” age group for both females (24.48%) and men (30.75%). Most of the cohort was without DM, hypertension, or hyperlipidemia. Base on the rule of NHIR in Taiwan, subjects who with the higher socioeconomic status (SS), the higher insurance premium should be pay. The insured amount of NHIR depends on the individual’s SS. Therefore, the SS can be estimated from the insurance amount and a higher-than-average insured amount was defined as high SS in the research.

The IHD incidence rate during follow-up for 12 years was 9.2% in men and 4.8% in women in the SLE group, compared to 2.3% and 3.5%, respectively, in the control group. Exposure to air pollution was slightly higher in females in both the SLE and control groups.

**Table 1:** Distributions of demographic and clinical comorbid status in study cohorts

				SLE					Control			
			Male			Female			Male			Female
		n/mean	(%)/sd		n/mean	(%)/sd		n/mean	(%)/sd		n/mean	(%)/sd
***In 2006***												
Sex		1301	27		3541	73		5196	27		14,087	73
Age		49.91	18.78		46.1	16.2		43.58	16.92		42.77	16.43
	20–29	191	14.68		542	15.31		1160	22.17		3260	23.09
	30–39	222	17.06		645	18.22		1048	20.02		2929	20.74
	40–49	263	20.22		733	20.7		1093	20.88		2922	20.69
	50–59	225	17.29		754	21.29		861	16.38		2349	16.61
	Above 60	400	30.75		867	24.48		1077	20.55		2669	18.88
DM	no	1287	98.92		3520	99.41		5032	96.44		13,675	96.93
	yes	14	1.08		21	0.59		207	3.56		454	3.07
HT	no	1277	98.16		3502	98.9		4823	92.42		13,202	93.58
	yes	24	1.84		39	1.1		416	7.58		927	6.42
HL	no		100		3514	99.24		5087	97.5		13,837	98.08
	yes				27	0.76		151	2.5		292	1.92
SS		26,850			25625.37			26205.46			23822.19	
***In 2018***												
IHD	no	1492			3044			5494			13,265	
	yes	152			154			130			479	
Air	CO	5.16			5.7			5.32			5.64	
pollution	NO_2_	188.87			208.5			194.04			206.06	
	O_3_	313.3			342.3			335.17			349.42	
	PM_10_	563.81			611.87			603.34			629.69	
	PM_2.5_	327.88			355.53			344.2			359.81	
	SO_2_	38.9			42.06			43.11			44.97	

SLE: Systemic Lupus Erythematosus IHD: Ischemic heart disease DM: Diabetes Mellitus.

HT: Hypertension HL: Hyperlipidemia SS: Socioeconomic status.

CO: carbon monoxide NO_2_: nitrogen dioxide O_3_: ozone.

PM_10_: particulate matter 10 PM_2.5_: particulate matter 2.5 SO_2_: sulfur dioxide.

Table 2 shows Pearson’s correlation of air pollution indices, CO, NO_2_, O_3_, PM_10_, PM_2.5_, and SO_2_. All the air pollution indices were significantly correlated (*p* < 0.01). Correlations of O_3_ with CO (r = 0.968, *p* < 0.01), and PM_10_ with PM_2.5_(r = 0.951, *p* < 0.01) were above 0.9. The lowest correlation was found between PM_10_ and NO_2_ (r = 0.408, *p* < 0.01). The presence of high correlations suggests that collinearity should be considered. We further defined the higher than average of each air pollution index as the “high exposure group” and others as the “low exposure group” for the Cox proportional risk model of time-dependent covariance.

**Table 2:** Correlations among air pollution indices

	CO	NO_2_	O_3_	PM_10_	PM_2.5_	SO_2_
CO	1	0.968^**^	0.544^**^	0.465^**^	0.449^**^	0.652^**^
NO_2_		1	0.437^**^	0.408^**^	0.423^**^	0.685^**^
O_3_			1	0.821^**^	0.768^**^	0.793^**^
PM_10_				1	0.951^**^	0.739^**^
PM_2.5_					1	0.743^**^
SO_2_						1

** *p* < 0.01

CO: carbon monoxide NO_2_: nitrogen dioxide O_3_: ozone

PM_10_: particulate matter 10 PM_2.5_: particulate matter 2.5 SO_2_: sulfur dioxide

**Fig. 2:**
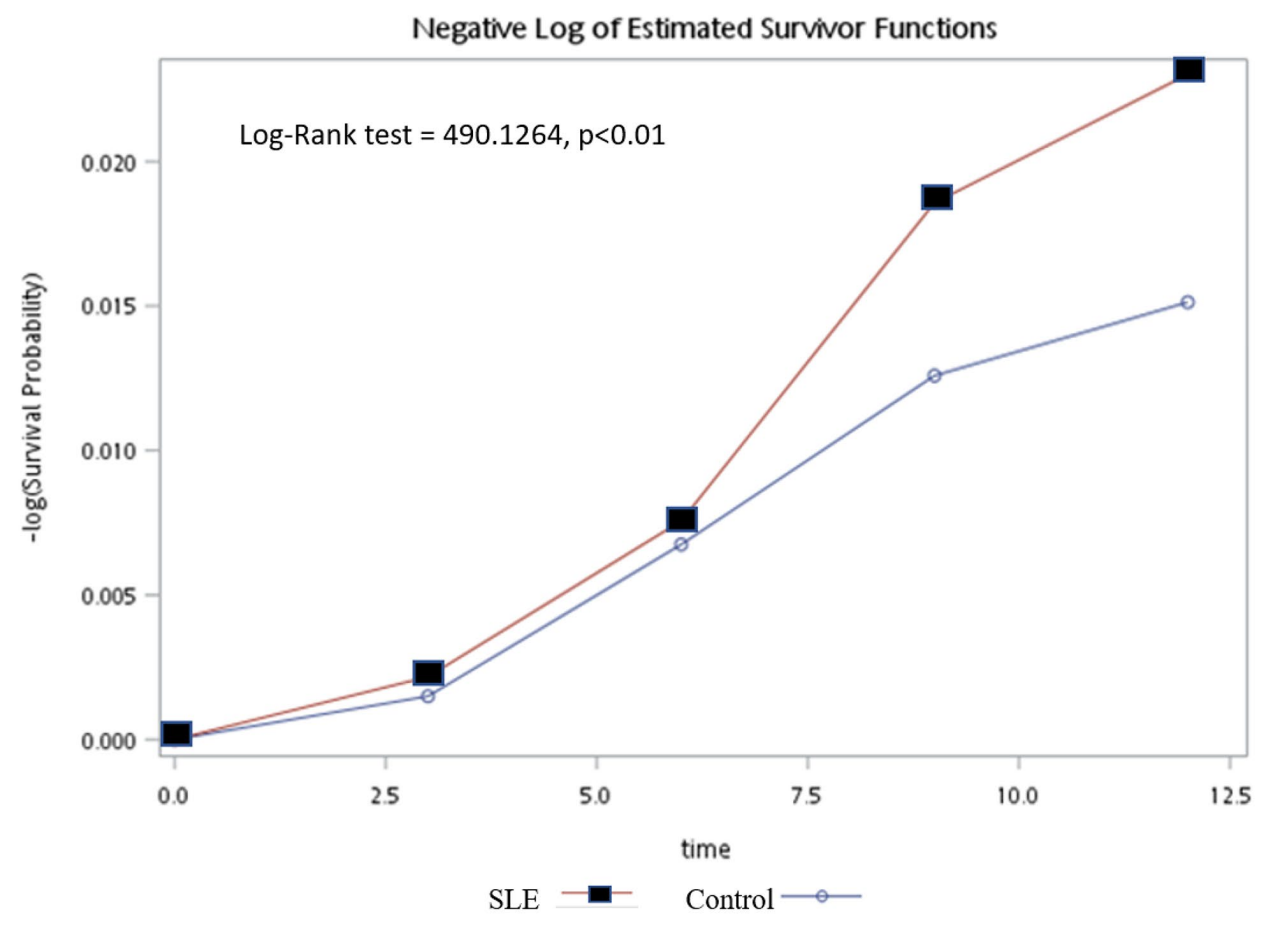
The probability of ischemic heart diseases incidence for the SLE and control group

The life-table method was used to measure the difference in IHD incidence between the SLE and control groups (Table 3). The estimated hazard curves of those two groups were tested using the log-rank test. Our study showed a statistically significant difference in IHD incidence between the SLE and control groups (log-rank test, *p* < 0.001). During the follow-up period, the risk of IHD incidence was higher in the SLE group (Fig. 2). The difference in IHD incidence between the SLE and control group peaked between the 6th and 9th years, during which period the hazard ratio of the SLE group was 2.42 times that of the control group (Table 3).

**Table 3:** Duration of progression to IHD in the SLE and control group

Group	Interval (year)	Log-Rank test	Survival (%)	Failure (%)	Survival SE (‰)	Evaluated at the midpoint of the interval	
						Hazard	Hazard SE
SLE		490.1264*					
	0–3		1	0	0	0.001015	0.000089
	3–6		99.7	0.30	0.266	0.002568	0.000142
	6–9		98.9	1.07	0.499	0.005722	0.000215
	9–12		97.3	2.75	0.796	0.002551	0.000146
	12		96.5	3.49	0.896	.	.
Control							
	0–3		1	0	0	0.000618	0.000033
	3–6		99.8	0.19	0.099	0.001988	0.000060
	6–9		99.2	0.78	0.203	0.002360	0.000066
	9–12		98.5	1.48	0.280	0.001004	0.000043
	12		98.2	1.77	0.307	.	.

* *p* < 0.01

We further evaluated whether comorbidities and air pollution exposure impacted IHD incidence by stratifying the air pollution exposure into two segments (high segment ≥ mean, low segment < mean) in SLE subjects (Table 4) and control subjects (Table 5). The Cox proportional risk model of time-dependent covariance analyses revealed that sex, age, CO, NO_2_, PM_10_, and PM_2.5_ were significantly related to developing IHD in both the SLE and control groups. The highest risk of developing IHD was identified on PM_10_ exposure in SLE subjects (HR = 66.197, *p* < 0.01) and in the control group (HR = 108.945, *p* < 0.01). The risk of developing IHD was significantly higher in the male cohort than the female cohort in both the SLE group (HR = 1.817, *p* < 0.01) and the control group (HR = 1.609, *p* < 0.01). In the control group, having diabetes was also associated with a 1.57-fold increase in IHD incidence (*p* < 0.001). Compared with the SLE group, the prevalence of diabetes was not significantly related to IHD incidence.

**Table 4:** The HR of development to IHD in the SLE group under air pollution (N = 4842)

		Parameter Estimate	SE	Chi-Square	HR	95% HR Confidence
Sex	Male	0.59724	0.17771	11.2947*	1.817	1.283, 2.574
	Female *(ref)*				1	
Age		0.10032	0.0068	217.62*	1.106	1.091, 1.12
DM	yes	-0.15135	1.01579	0.0222	0.86	0.117, 6.294
	*no(ref)*				1	
HT	yes	0.33328	0.46448	0.5149	1.396	0.562, 3.468
	*no(ref)*				1	
HL	yes	0.19407	0.23181	0.7009	1.214	0.771, 1.912
	*no(ref)*				1	
SS	High	0.56996	1.00916	0.319	1.768	0.245, 12.78
	*Low(ref)*				1	
CO	high	2.56001	0.51353	24.8512*	12.936	4.728, 35.393
	low*(ref)*				1	
NO_2_	high	3.89938	0.43186	81.5272*	49.372	21.178, 115.101
	low*(ref)*				1	
O_3_	high	-0.0731	0.04957	2.1761	0.929	0.843, 1.024
	low*(ref)*				1	
PM_10_	high	4.19263	0.51048	67.4557*	66.197	24.34, 180.035
	low*(ref)*				1	
PM_2.5_	high	3.54241	0.39236	81.5125*	34.55	16.013, 74.546
	low*(ref)*				1	
SO_2_	high	0.0304	0.19678	0.0239	1.031	0.701, 1.516
	low*(ref)*				1	

h: Hazard ratio SE: Standard error *p < 0.01

SLE: Systemic Lupus Erythematosus IHD: Ischemic heart disease DM: Diabetes Mellitus

HT: Hypertension HL: Hyperlipidemia SS: Socioeconomic status

CO: carbon monoxide NO_2_: nitrogen dioxide O_3_: ozone

PM_10_: particulate matter 10 PM_2.5_: particulate matter 2.5 SO_2_: sulfur dioxide

**Table 5:** The HR of development to IHD in the control group under air pollution (N = 19,368)

		Parameter Estimate	SE	Chi-Square	HR	95% HR Confidence
Sex	Male	0.4759	0.09046	27.6763*	1.609	1.348, 1.922
	Female *(ref)*				1	
Age		0.11337	0.00348	1058.51*	1.12	1.112, 1.128
DM	yes	0.45079	0.15837	8.102*	1.57	1.151, 2.141
	*no(ref)*				1	
HT	yes	-0.0383	0.12335	0.0962	0.962	0.756, 1.226
	*no(ref)*				1	
HL	yes	0.18626	0.23133	0.6483	1.205	0.766, 1.896
	*no(ref)*				1	
SS	High	0.27921	0.45263	0.3805	1.322	0.544, 3.21
	*Low(ref)*				1	
CO	high	3.0434	0.32109	89.8381*	20.976	11.179, 39.359
	low*(ref)*				1	
NO_2_	high	4.0207	0.26526	229.757*	55.74	33.142, 93.747
	low*(ref)*				1	
O_3_	high	-0.0428	0.02476	2.9802	0.958	0.913, 1.006
	low*(ref)*				1	
PM_10_	high	4.69084	0.35732	172.345*	108.945	54.083, 219.459
	low*(ref)*				1	
PM_2.5_	high	3.43357	0.20312	285.757*	30.987	20.811, 46.14
	low*(ref)*				1	
SO_2_	high	4.63071	0.38239	146.652*	102.587	48.485, 217.061
	low*(ref)*				1	

h: Hazard ratio SE: Standard error *p < 0.01

SLE: Systemic Lupus Erythematosus IHD: Ischemic heart disease DM: Diabetes Mellitus

HT: Hypertension HL: Hyperlipidemia SS: Socioeconomic status

CO: carbon monoxide NO_2_: nitrogen dioxide O_3_: ozone

PM_10_: particulate matter 10 PM_2.5_: particulate matter 2.5 SO_2_: sulfur dioxide

## Discussion

This cohort study analyzed the risk of developing IHD among newly diagnosed SLE subjects from 2006 to 2018. The study results revealed three findings. First, during the follow-up period, the risk of IHD incidence was higher in the SLE group. The difference in IHD incidence between the SLE and control groups peaked between the 6th and 9th year after SLE diagnosis. Second, the peak hazard ratio of IHD in the SLE population occurred during this period. Third, sex, age, CO, NO_2_, PM_10_, and PM_2.5_ significantly contributed to the progression to IHD in SLE patients; PM_10_ was the highest risk factor for both the SLE and control groups.

It is well known that subjects with atherosclerosis are susceptible to suffering from IHD, and previous studies have documented that the hazard of atherosclerosis is higher in SLE subjects than in the general population [11]. Some studies have also indicated that IHD incidence increases in subjects with SLE [3, 12, 13]. The disturbance of the immune system and inflammation could promote atherosclerosis and further contribute to IHD [14]. In the present study, the risk of progression to IHD after SLE diagnosis in the SLE group was higher than in the control group, which was consistent with the previous literature.

We found that the highest hazard ratio of IHD occurred in the 6th -9th year after SLE diagnosis and that the hazard ratio in the SLE group was 2.42 times that of the control group. A previous study indicated that subjects with SLE had a higher risk of suffering from cardiovascular diseases compared to the general population, reporting a similar range as in our study (HR 2.67 [95% CI 2.38–2.99]) [15]. In contrast, another study found that the relative risk of myocardial infarction was between five to eight times greater in SLE subjects compared with the general population [11]. However, there was a difference in the study populations, as we recruited not only myocardial infarction patients but also angina patients. The severity and lethality were more serious in myocardial infarctions than in angina, which might contribute to the difference in results.

Our results indicated that the highest HR of IHD occurred in the 6th -9th year after SLE diagnosis and that HR decreased after the 9th year. Previous studies showed that the traditional pathological mechanism could not explain the progression of cardiovascular disease induced by SLE. Vascular events may result from several pathophysiologic mechanisms, including atherosclerosis, primarily thrombotic, and ongoing inflammation [16]. The primary pathogenesis of IHD in the SLE group and the general population is that inflammation due to SLE might strongly contribute to developing IHD. In the absence of immune system problems, traditional risk factors might influence the general population suffering from IHD. Therefore, the IHD incidence differed between SLE subjects and the general population. Inflammation increased with age in the subjects diagnosed with SLE, and theoretically, the risk of progression to IHD was also elevated. Although this study showed that SLE subjects had a higher risk of developing IHD than the control group, the highest HR occurred in the 6th to 9th year after SLE diagnosis. However, the HR gradually declined after the 9th year. A previous study showed a significant difference in the prevalence of cardiovascular disease in subjects of different ethnicities [17], which might contribute to the discrepancy in results between our study and previous research. In addition, since the quality of the medical system in Taiwan is excellent and the case management system for SLE is quite mature, the SLE population may have improved their self-care skills and knowledge in spite of inflammation increasing over the years. This may have prevented an increase in the difference in the HR of IHD between the case and control groups.

Women with diabetes have a greater relative risk of cardiovascular disease compared with their male counterparts [18]. In the present study, diabetes was a significant risk factor in the control subjects who developed IHD, but not a significant risk factor for developing IHD in the SLE group, which is inconsistent with previous findings. Differences in study populations and ethnicity might explain this difference.

A previous study demonstrated a 50 times greater risk of myocardial infarction in female patients with SLE aged 35–44 years than in women of similar age without SLE in a population-based sample [19]. In our study, female patients in the control group aged 30–59 years were associated with a lower risk of IHD (HR < 1); this age group may include women who have not experienced menopause, and estrogen might thus exert a protective effect on their cardiovascular system. The results of our study are consistent with previous findings that estrogen levels associated with the menstrual cycle may contribute to the delayed onset of cardiovascular diseases in women compared with men [18]. However, patients with SLE from the same age group were at a higher risk of IHD (HR 1.27–2.67), indicating that SLE might increase the susceptibility of this population to IHD.

## Conclusions

Subjects with SLE were at a higher risk of IHD, especially those in the 6th to 9th year after SLE diagnosis. Since the highest risk for developing IHD in SLE patients is during this period, it is recommended that advanced cardiac health examinations be included in the health insurance benefits for SLE patients in the 5th to 9th year after SLE diagnosis. At the same time, case managers should make sure that education on diet and exercise is included in the health education plan for first-diagnosed SLE patients. The health education plan could promote a healthier lifestyle for SLE patients to reduce the risk of atherosclerosis or IHD.

This retrospective study used a secondary database search of the NHIR to explore the risk of IHD in first-diagnosed SLE patients. A limitation of this approach was the absence of data on items such as psychosocial issues, clinical measurements, and lifestyle. Future related research on clinical trials should include these data. The results of this study provide a reference for the next phase of clinical trials.

This study has some limitations. First, using the health insurance database, we recruited the subjects only by the ICD-9 code. This database did not include information such as psychosocial information (depression, anxiety, A-type, etc.), health promotion data (smoking and drinking), family history (hereditary disease), and medication adherence. Second, residence status was likely partly inaccurate as subjects might move house. Finally, smoking is one of the risk factors for atherosclerosis. Both direct and second-hand smokers are at risk of nicotine exposure. Unlike the United States and Europe, most areas in Taiwan have a high population density, increasing exposure to a smoking environment even for non-smokers. In the future, relevant clinical studies should identify whether carbon monoxide in exhaled breath can be used in non-smokers to identify smoking status.

## Data Availability

All data generated or analyzed during this study are included in this published article. The data are available from the first author on reasonable request. The first author is Pei-Yun Chen (e-mail: peiyun0203@gmail.com).

## Abbreviations

SLE: Systemic Lupus Erythematosus
IHD: Ischemic heart disease
DM: Diabetes Mellitus
HT: Hypertension
HL: Hyperlipidemia
CO: carbon monoxide
NO_2_: nitrogen dioxide
O_3_: ozone
PM_10_: particulate matter 10
PM_2.5_: particulate matter 2.5
SO_2_: sulfur dioxide
